# Supplementary figures and images for: Representative Diatom and Coccolithophore Species Exhibit Divergent Responses throughout Simulated Upwelling Cycles
Source: mSystems. 2021 Mar 30;6(2):e00188-21. doi: 10.1128/mSystems.00188-21 (PMC8546972; doi:10.1128/mSystems.00188-21)

# *C. decipiens*

Fe-replete

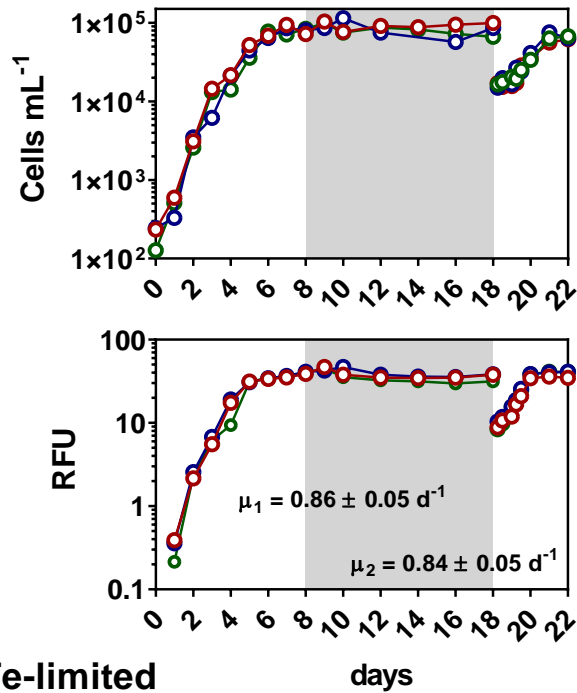

Fe-limited

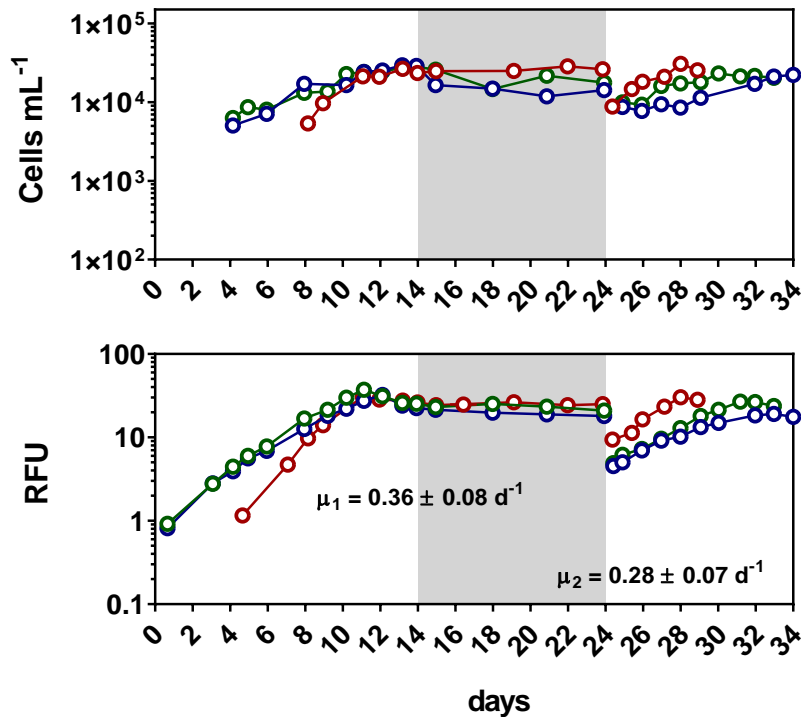

# *E. huxleyi*

Fe-replete

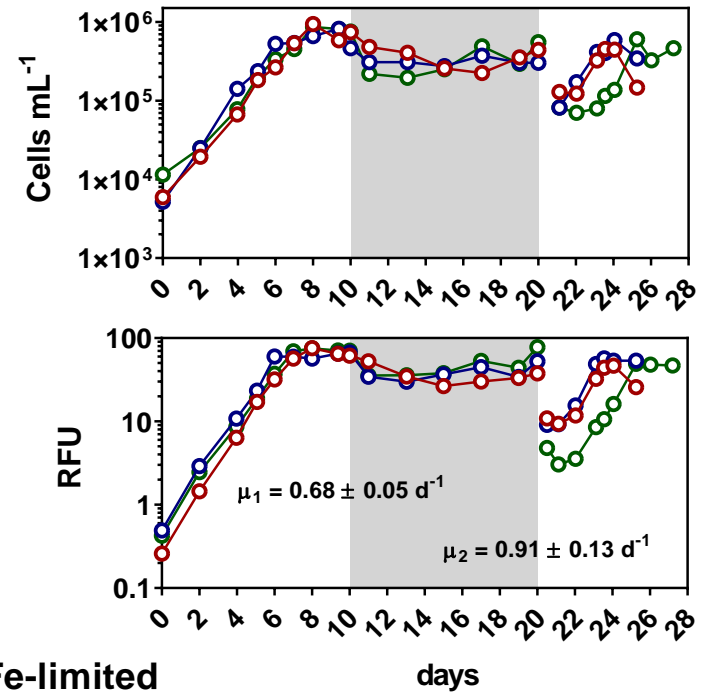

Fe-limited

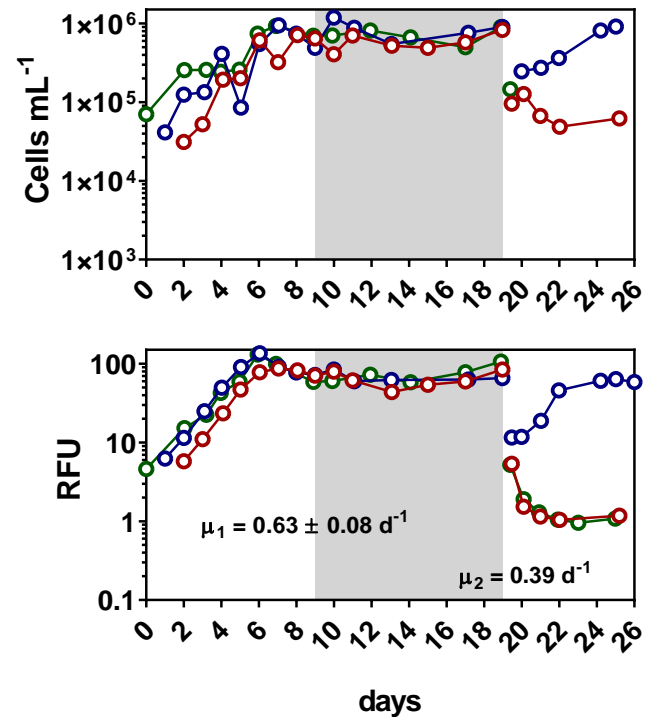

Supplement: FIG S1 [file msystems.00188-21-sf001.pdf]

# *C. decipiens*

Fe-replete

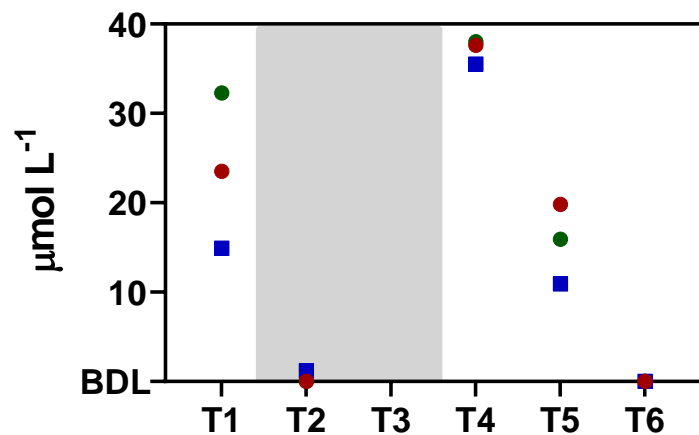

Fe-limited

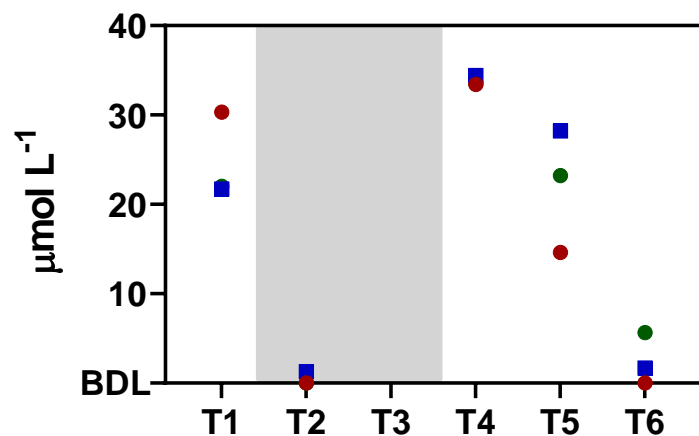

Time point

# *E. huxleyi*

Fe-replete

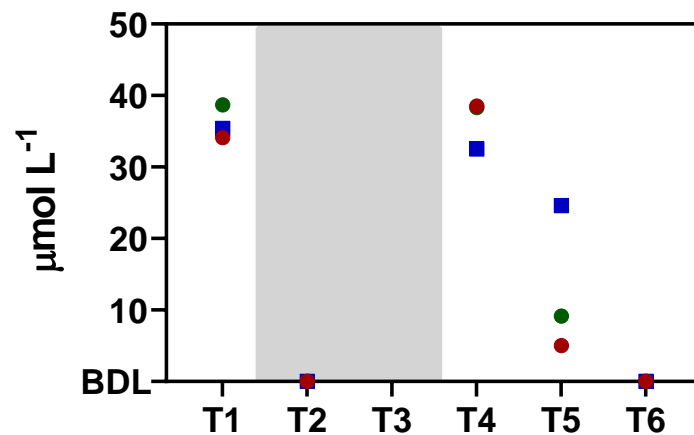

Fe-limited

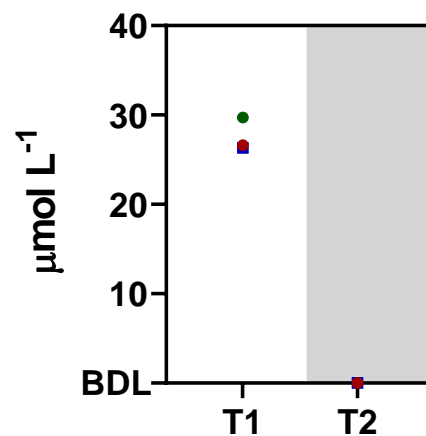

Time point

Supplement: FIG S2 [file msystems.00188-21-sf002.pdf]

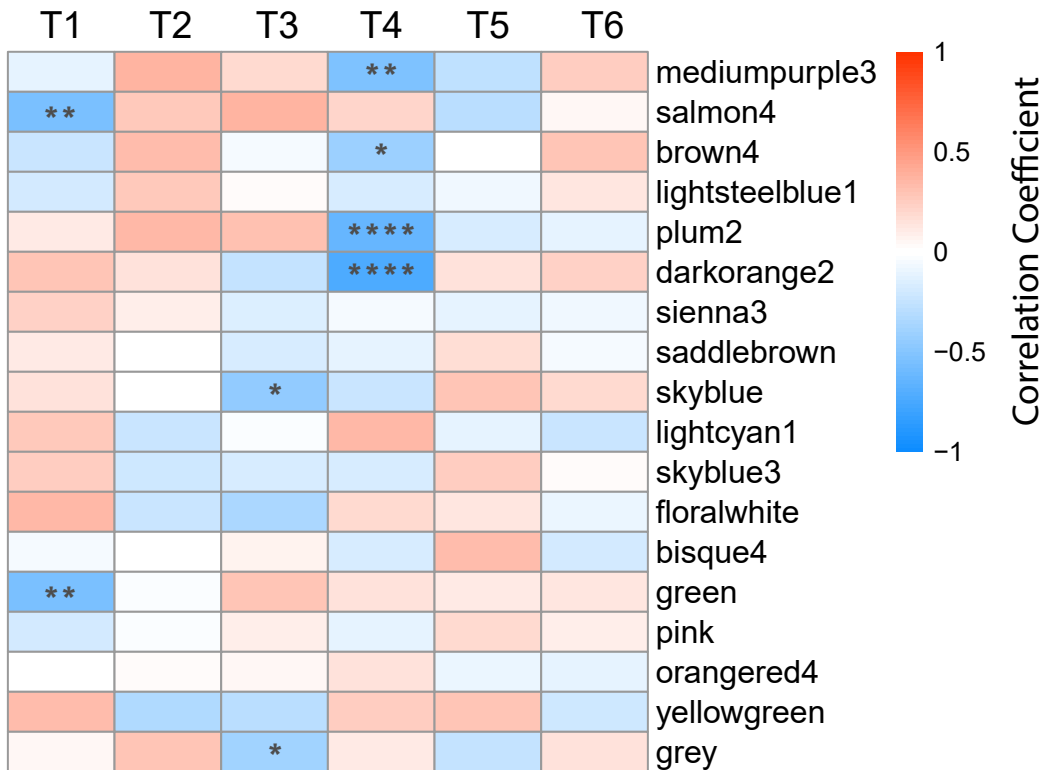

Supplement: FIG S3 [file msystems.00188-21-sf003.pdf]

# *C. decipiens*

Fe-replete

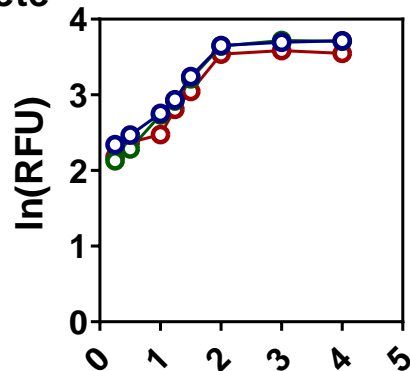

Fe-limited

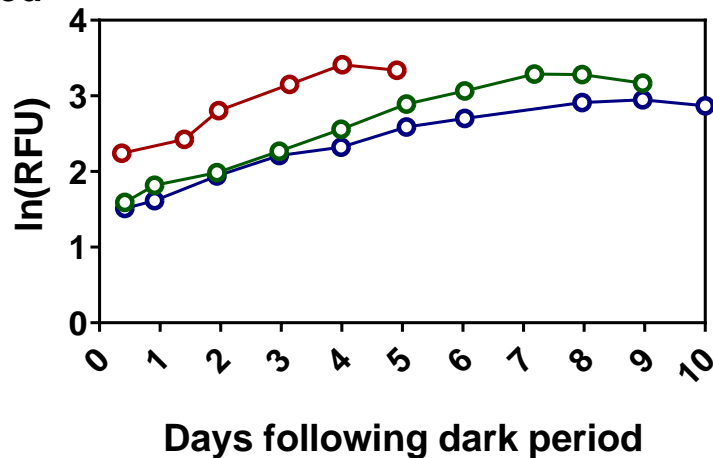

# *E. huxleyi*

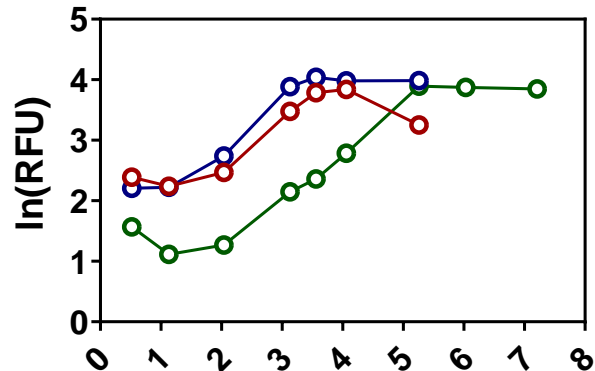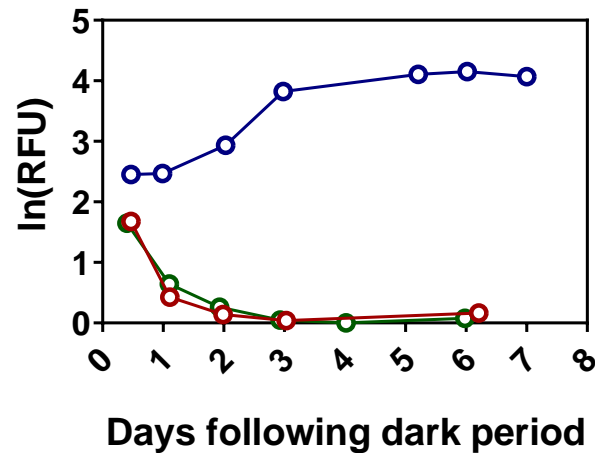

Supplement: FIG S4 [file msystems.00188-21-sf004.pdf]

Dark Period

## *C. decipiens*

## *E. huxleyi*

5 days

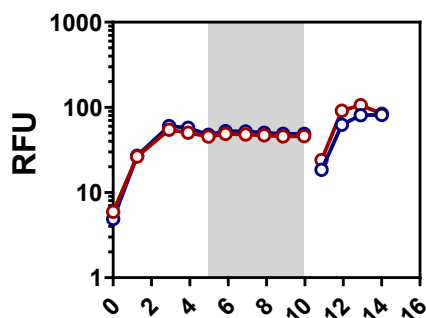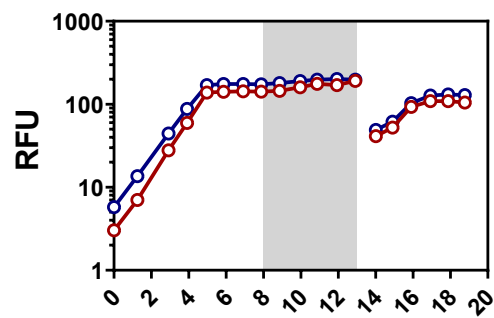

10 days

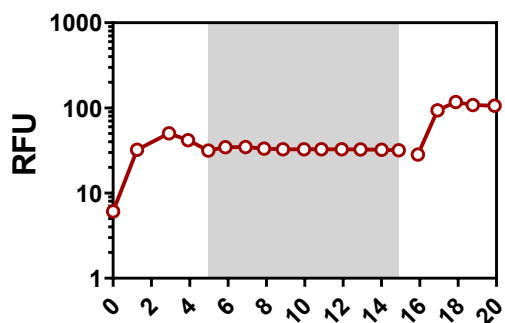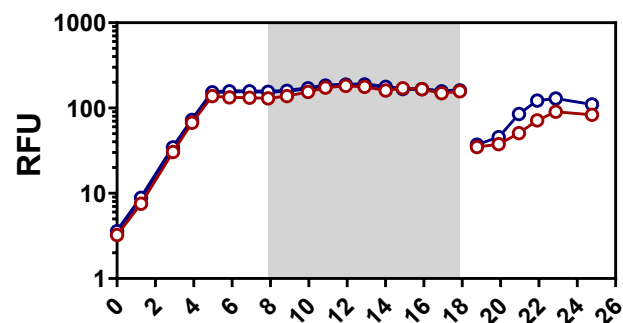

15 days

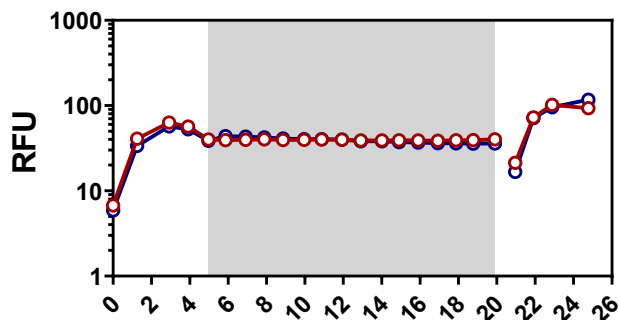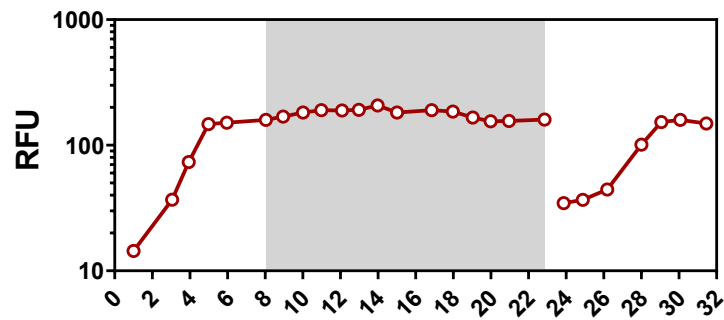

20 days

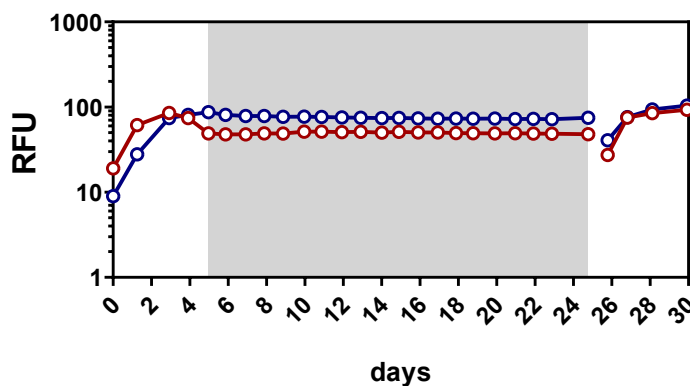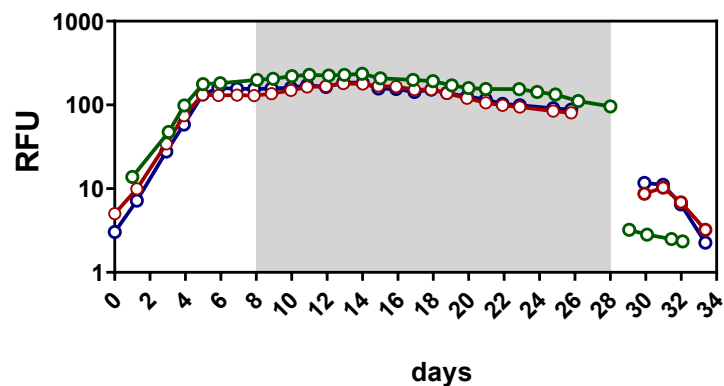

Supplement: FIG S5 [file msystems.00188-21-sf005.pdf]

## *C. decipiens*

**A**

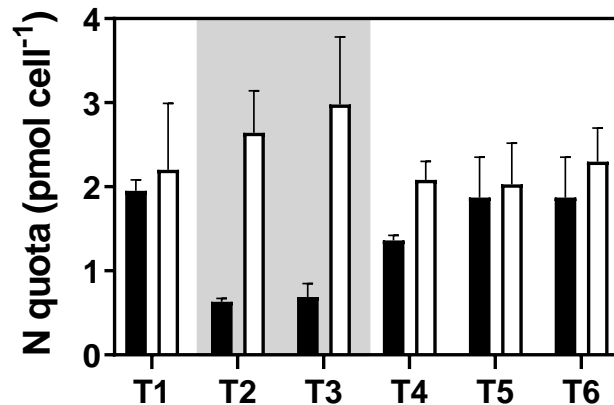

**B**

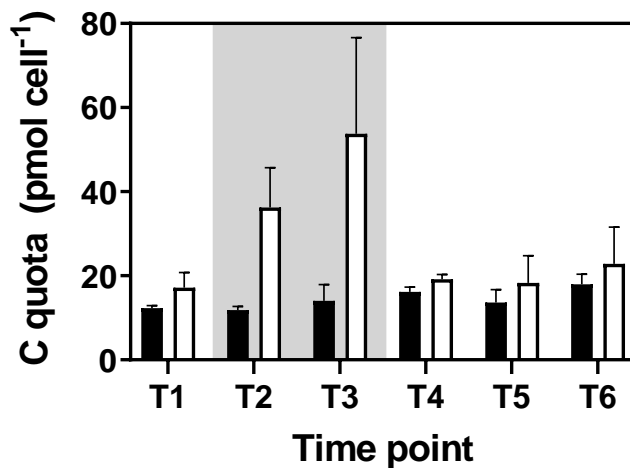

## *E. huxleyi*

**C**

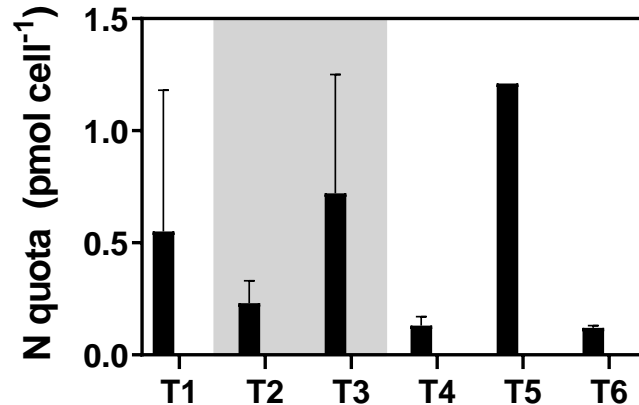

**D**

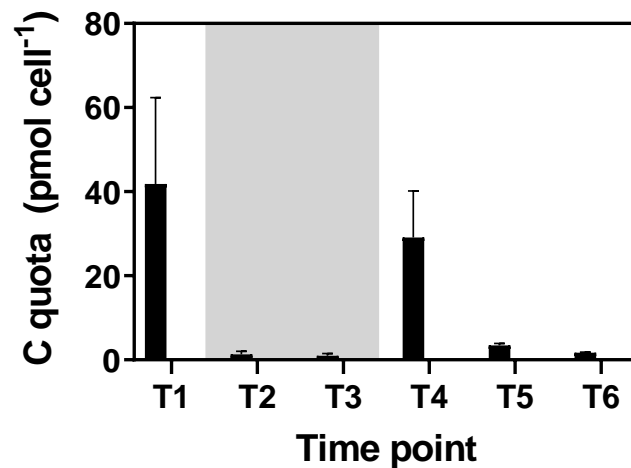

■ Fe-replete    □ Fe-limited

Supplement: FIG S6 [file msystems.00188-21-sf006.pdf]

## *C. decipiens*

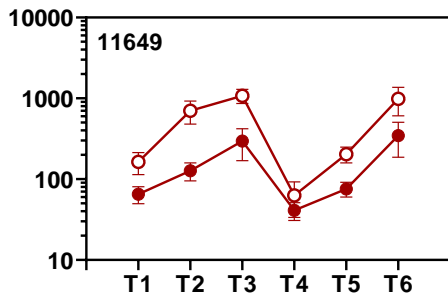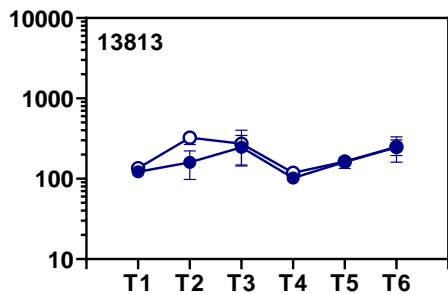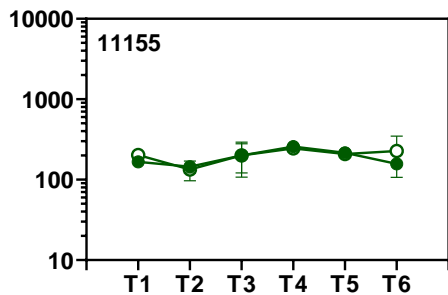

Time point

## *E. huxleyi*

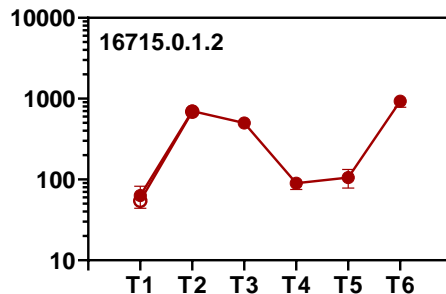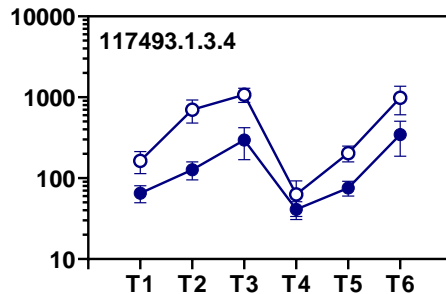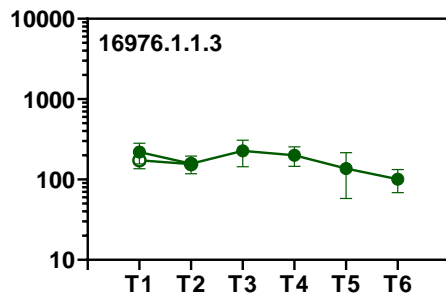

Time point

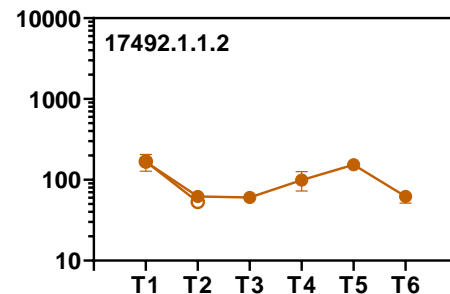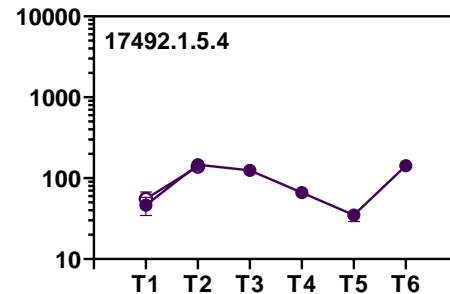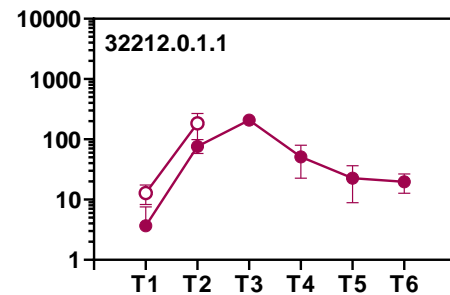

Time point

Supplement: FIG S7 [file msystems.00188-21-sf007.pdf]

**A*****C. decipiens***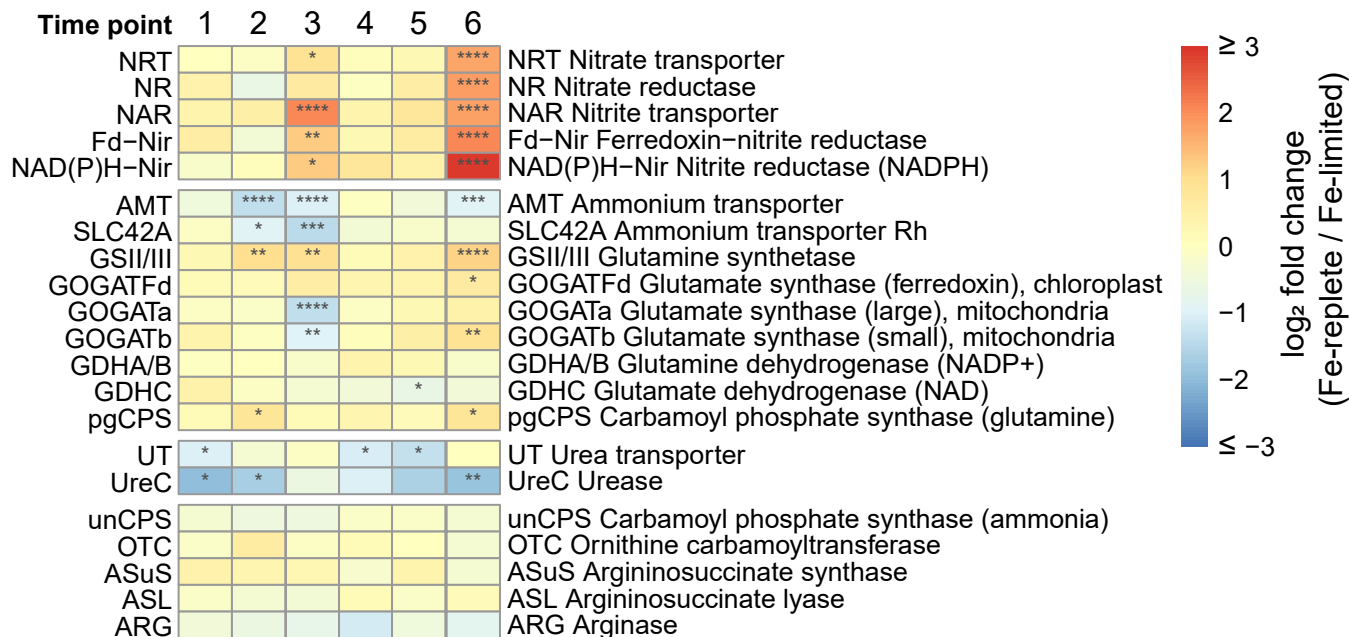**B*****E. huxleyi***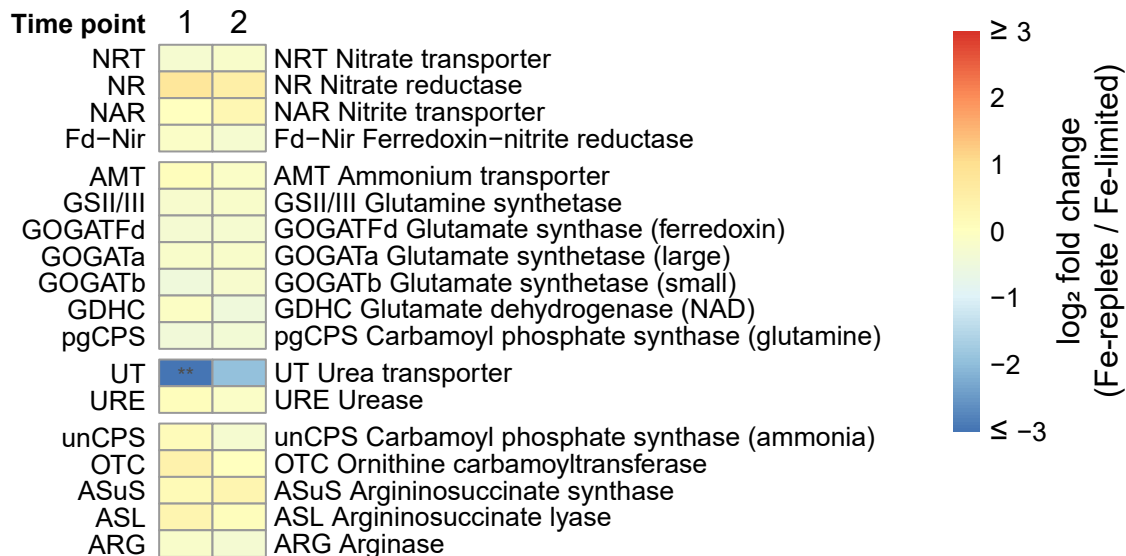

Supplement: FIG S10 [file msystems.00188-21-sf010.pdf]
