# Supplementary material for: Representative Diatom and Coccolithophore Species Exhibit Divergent Responses throughout Simulated Upwelling Cycles
Source: mSystems. 2021 Mar 30;6(2):e00188-21. doi: 10.1128/mSystems.00188-21 (PMC8546972; doi:10.1128/mSystems.00188-21)
Supplement: FIG S8 [file msystems.00188-21-sf008.pdf]

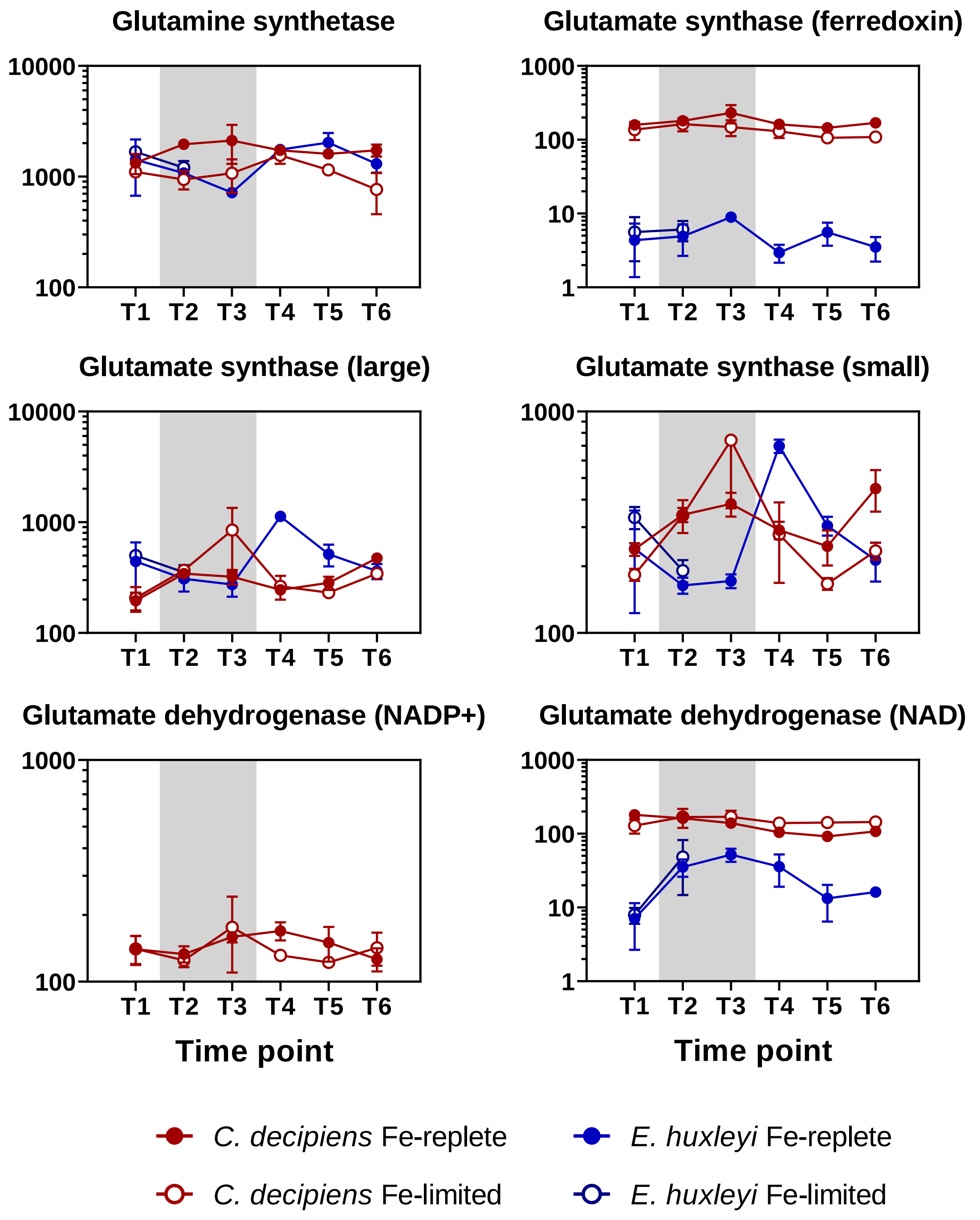

Glutamine synthetase
Glutamate synthase (ferredoxin)
Glutamate synthase (large)
Glutamate synthase (small)
Glutamate dehydrogenase (NADP+)
Glutamate dehydrogenase (NAD)
10000
1000
100
10
1
T1 T2 T3 T4 T5 T6
Time point
C. decipiens Fe-replete
C. decipiens Fe-limited
E. huxleyi Fe-replete
E. huxleyi Fe-limited
