# Supplementary material for: Representative Diatom and Coccolithophore Species Exhibit Divergent Responses throughout Simulated Upwelling Cycles
Source: mSystems. 2021 Mar 30;6(2):e00188-21. doi: 10.1128/mSystems.00188-21 (PMC8546972; doi:10.1128/mSystems.00188-21)
Supplement: FIG S9 [file msystems.00188-21-sf009.pdf]

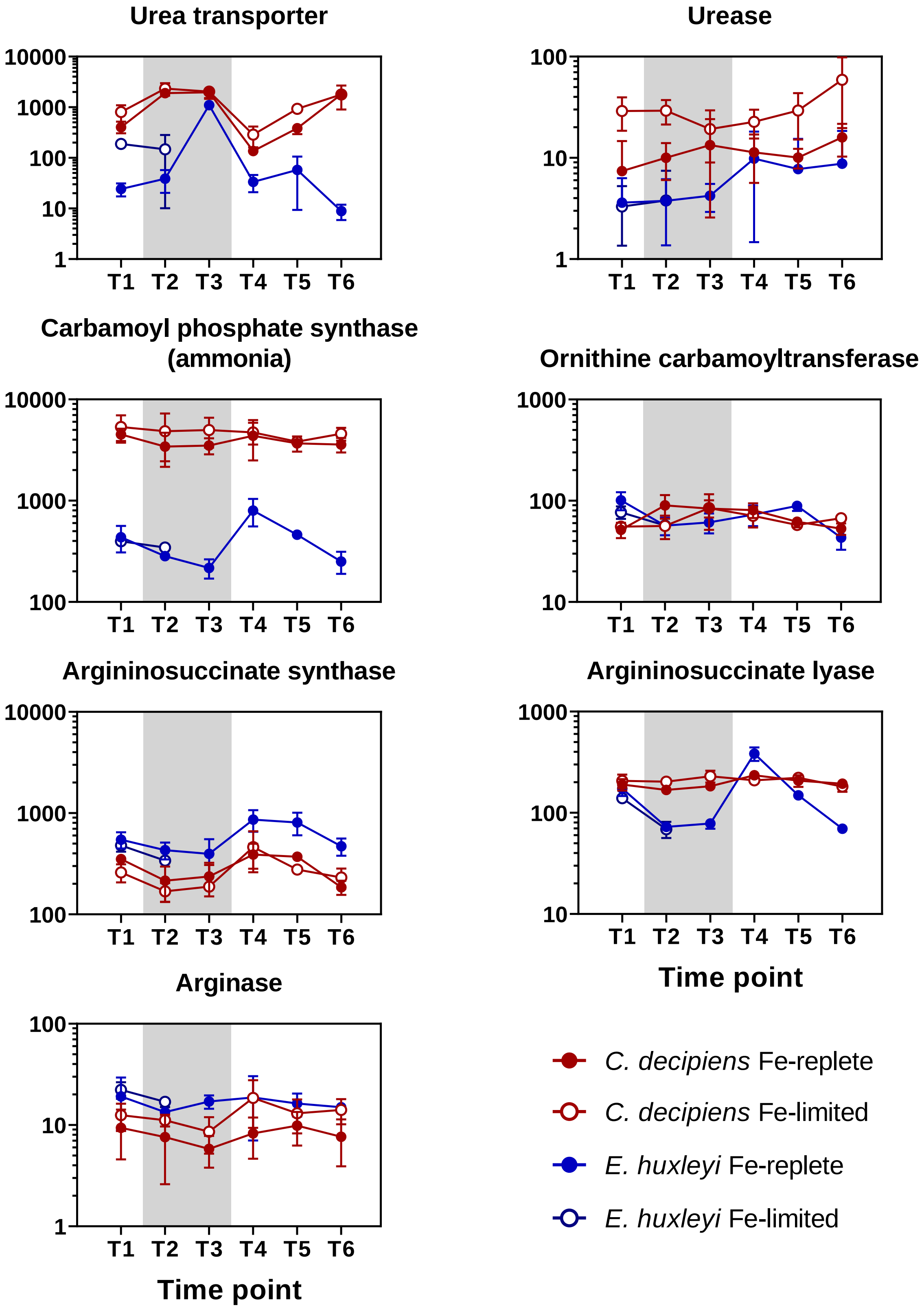

Urea transporter
10000
1000
100
10
1
T1 T2 T3 T4 T5 T6
Urease
100
10
1
T1 T2 T3 T4 T5 T6
Carbamoyl phosphate synthase (ammonia)
10000
1000
100
T1 T2 T3 T4 T5 T6
Ornithine carbamoyltransferase
1000
100
10
T1 T2 T3 T4 T5 T6
Argininosuccinate synthase
10000
1000
100
T1 T2 T3 T4 T5 T6
Argininosuccinate lyase
1000
100
10
T1 T2 T3 T4 T5 T6
Time point
Arginase
100
10
1
T1 T2 T3 T4 T5 T6
Time point
C. decipiens Fe-replete
C. decipiens Fe-limited
E. huxleyi Fe-replete
E. huxleyi Fe-limited
